# Supplementary material for: Content-rich biological network constructed by mining PubMed abstracts
Source: BMC Bioinformatics. 2004 Oct 8;5:147. doi: 10.1186/1471-2105-5-147 (PMC528731; doi:10.1186/1471-2105-5-147)
Supplement: Additional File 2 — The original results of the above study (non-essential files are deleted to keep the file size under the limit set by BMC bioinformatics). [file 1471-2105-5-147-S2.bz2 › chilibotAdditionalFile2/dip05/278ID2497053E1101/html/JUN_FOSL1.html]

 


 **JUN** and **FOSL1** 
  
Found 404 abstracts in PubMed, retrieved 05.  
 

 What does Google say? 
 PDF only 
| .edu only 

---

**Interactive relationship** (e.g. stimulation, inhibition, etc)

**Neutral relationship**- Here, the major cis regulatory elements that control IFN gamma inducible NOS2 gene transcription in human lung epithelial cells are identified as composite response elements that bind signal transducer and activator of transcription 1 STAT 1 and activator protein 1  [ **JUN** ]  AP 1  [ **JUN** ] , which is comprised of c Fos, Fra  [ **FOSL1** ]  2, c  **Jun** , and JunD.  Ref: 12788789 Am J Physiol Lung Cell Mol Physiol, 2003

**Non-interactive relationship** (e.g. studied together, co-existance, homology, etc.)

- MATERIAL AND METHODS From cDNA microarray screening of those specimens, several gene clusters, including Nos2 and the transcription factors Fos,  **Fosl1** ,  **Jun**  and Nfkb1, were identified as possible upstream regulators of Na transport protein expression.  Ref: 12875578 Acta Otolaryngol, 2003
- Among several immediate early gene products, only  **Jun**  B and Fra 1  [ **FOSL1** ]  protein levels were increased by LNT, and ginsenoside Rd attenuated  **Jun**  B and Fra 1  [ **FOSL1** ]  protein levels induced by LNT.  Ref: 12785733 Arch Pharm Res, 2003
- Our results suggest that ginsenoside Rd exerts an inhibitory action against NO production via blocking phosphorylation of ERK, in turn, suppressing immediate early gene products such as  **Jun**  B and Fra 1  [ **FOSL1** ]  in C6 glioma cells.  Ref: 12785733 Arch Pharm Res, 2003
- To this end, we studied the kinetics of serum stimulated transcription of c fos, c  **jun** , fra 1  [ **FOSL1** ] , egr 1, and cyclinD1 genes, as well as the effects of sodium butyrate, an inhibitor of histone deacetylase activity, on transcription of these genes in normal REF cells and transformants E1A ras.  Ref: 12804589 Biochem Biophys Res Commun, 2003
- Gene expression for Fos was suppressed at both times, while that for  **Fosl1**  and  **Jun**  was augmented at 12 h and suppressed at 48 h.  Ref: 12875578 Acta Otolaryngol, 2003
- In a Gal4 fusion assay, Pdcd4 specifically inhibited activation of c  **Jun**  and c Fos activation domains, but did not inhibit activation of JunB, JunD, Fra 1  [ **FOSL1** ] , or Fra  [ **FOSL1** ]  2.  Ref: 12802278 Oncogene, 2003
